# Development and Utilization of a Custom PCR Array Workflow: Analysis of Gene Expression in *Mycoplasma genitalium* and Guinea Pig (*Cavia porcellus*)

**DOI:** 10.1007/s12033-014-9813-6

**Published:** 2014-10-31

**Authors:** Ronald L. Veselenak, Aaron L. Miller, Gregg N. Milligan, Nigel Bourne, Richard B. Pyles

**Affiliations:** 1 Department of Microbiology and Immunology, University of Texas Medical Branch, 301 University Blvd, Galveston, TX 77555-0436 USA; 2 Department of Pediatrics, University of Texas Medical Branch, 301 University Blvd, Galveston, TX 77555-0436 USA

**Keywords:** PCR array, Gene expression, Transcription, *Mycoplasma genitalium*, *Cavia porcellus*, Guinea pig, Immune response

## Abstract

Transcriptome analysis is a powerful tool for evaluating molecular pathways central to maturation of specific biological processes and disease states. Recently, PCR-based arrays have supplemented microarray and RNA-seq methodologies for studying changes in gene expression levels. PCR arrays are a more cost efficient alternative, however commercially available assemblies are generally limited to only a few more widely researched species (e.g., rat, human, and mouse). Consequently, the investigation of emerging or under-studied species is hindered until such assays are created. To address this need, we present data documenting the success of a developed workflow with enhanced potential to create and validate novel RT-PCR arrays for underrepresented species with whole or partial genome annotation. Utilizing this enhanced workflow, we have achieved a success rate of 80 % for first-round designs for over 400 primer pairs. Of these, ~160 distinct targets were sequence confirmed. Proof of concept studies using two unique arrays, one targeting the pathogenic bacterium *Mycoplasma genitalium* and the other specific for the guinea pig (*Cavia porcellus*), allowed us to identify significant (*P* < 0.05) changes in mRNA expression validated by subsequent qPCR. This flexible and adaptable platform provides a valuable and cost-effective alternative for gene expression analysis.

**Electronic supplementary material:**

The online version of this article (doi:10.1007/s12033-014-9813-6) contains supplementary material, which is available to authorized users.

## Introduction

For many novel emerging or under-studied organisms, the lack of assays to quantify and characterize proteins and phenotype necessitates the evaluation of nucleic acids. While partial or complete genomic sequence may be available for many of these organisms, the expertise and cost required for de novo sequencing or to develop assays for measuring transcription level changes can be a significant deterrent to the development of custom in-house reagents. The lack of demand for niche products to address under-studied or emerging organisms further diminishes the likelihood of commercial production of reagents.

Transcriptional analysis can yield important information about expression patterns for developmental processes, evolving gene regulation, and for highlighting differences between healthy and diseased states [1]. For the last few decades, gene expression analysis has been dominated by the use of microarrays. Recently, new approaches using next-generation sequencing (NGS) have led to the development of improved transcriptomic techniques such as RNA-seq [2, 3]. However, transcriptome analysis by either microarray or RNA-seq is currently widely utilized in a discovery role for large-scale gene expression studies [3] and transcript-level analyses made possible by both technologies have revolutionized molecular biology [2, 4]. Microarrays are especially useful to explore gene expression of multiple cellular pathways in parallel and these assays are widely available for a number of model organisms [3]. The newer RNA-seq technologies can also fill this role and additionally offer lower background signal over microarray techniques [3]. Coupled with the detection of transcripts that may be at very high or very low abundance levels [3], RNA-seq is quickly displacing microarrays as the preferential tool for gene expression analysis [5, 6]. While these methods have undoubtedly increased our understanding of transcriptomics, they require specialized equipment and expensive reagents, particularly with respect to RNA-seq, leading to increased costs per sample interrogated. While advances to these technologies have substantially reduced costs in recent years, a 20 million read RNA-seq reaction can be ~$1,000USD/sample increasing to nearly $3,000USD/sample for de novo sequencing (personal communication and web search). An additional concern surrounding RNA-seq is the introduction of bias at many steps during the process associated with technical inexperience, leading to unwanted variation across samples [1, 5, 7, 8].

Real-time reverse transcription PCR (RT-PCR) has been a widely used methodology for detecting transcriptional changes in mRNA [9, 10], and is the preferred method for validating both microarray and RNA-seq gene expression studies [3, 11]. Recently, this technique has been used to create PCR arrays for use in high-throughput gene profiling. These arrays combine ease of use, reliability, and reproducibility of RT-PCR into a more cost-effective method for screening large numbers of genes simultaneously [3] and so provide an attractive alternative to microarray or NGS methodologies. However, their utility is currently limited due to the availability of arrays for only a few of the more widely used research species. The restricted number of genes that may be analyzed simultaneously also presents another disadvantage to this methodology [3].

Optimal screening of broad transcriptional changes by RT-PCR requires a design approach that allows for up to 96 targets to be processed through a single set of PCR parameters. PCR primer design has been described previously for single target and multiplex designs [12] but required some enhancement to address the needs created by the transcriptional array. Our goal was to create a highly adaptable and cost-effective workflow with enhanced primer design, a broadly useful PCR program and algorithms for data analysis to facilitate gene expression analysis in under-studied organisms. As a result, we present a flexible PCR array development and validation method that creates adaptable screening tools to study changes in the transcriptome of any species with adequate genomic sequence. As proof of concept, we present the successful development and validation of two disparate arrays targeting the bacterial pathogen *Mycoplasma genitalium* (MG) and the commonly utilized guinea pig (*Cavia porcellus*), both of which currently lack commercially available assays for transcriptome analysis.

## Materials and Methods

### Optimized Thermocycling Protocol

To identify a thermocycling protocol central to the functionality of our array platform, we first tested four established protocols using primer pairs from our first-generation designs. These first evaluations targeted RNA species produced by the sexually transmitted bacterial pathogen MG. Specifically, type strain MG G37 genomic sequence (GenBank Accession: L43967) was used as a template to design 11 sets of forward and reverse primers for highly conserved genes within the bacterium using generic primer selection parameters. Each primer pair was used to amplify three distinct concentrations of purified MG genomic DNA covering a range of 10^5^–10^7^ copies per reaction. The resulting quantification cycle (*C*_q_) values [12], generated during SYBR green-based real-time PCR amplification, provided the opportunity to calculate the amplification efficiency of the 11 primer pairs using each of the four PCR thermocycling protocols. These preliminary studies identified a protocol that produced the most consistent PCR efficiencies across the 11 primer pairs. In addition, a melt temperature (*T*_m_) analysis was performed to provide information on the identity of the amplimer created in each reaction.

The optimal protocol identified by this initial evaluation was repeatedly modified by empirical testing of additional primer pairs to refine the thermocycling conditions. This resulted in a stable set of parameters to serve as the fundamental PCR protocol for our array system. The resultant protocol, designated as “KS”, was established as our standard and subsequently used to evaluate and refine primer design methods to maximize successful targeting of selected genes.

### Primer Design and Optimization

Primers were designed using Beacon Designer v7.91 (PREMIER Biosoft; Palo Alto, CA, USA) and regions of each gene were selected by prescreening to identify areas that were highly conserved based on alignments of all available sequences. Initial observations from data obtained during our thermocycling protocol validation studies suggested that primers composed of less than 40 % guanine/cytosine (G/C) content were more efficient with the optimized KS protocol, a finding that was confirmed through subsequent primer design and testing studies. Thus, for maximum compatibility with the array platform, the optimal primer should incorporate ≤40 % G/C content. Our results also correlated PCR amplimer length with overall primer efficiency success rates. The data from almost 200 primer pairs led us to conclude that optimal amplimer length should be between 70 and 200 base pairs (bp) and led to the primer design specifications described below. All subsequent primer design was undertaken using these parameters.

Forward and reverse oligonucleotide primers were designed with custom settings in Beacon Designer v7.91. Previously reported primer design approaches [12] provided the basis for the initial parameters that were then empirically optimized. Primer length was 18–24 nucleotides and PCR amplimers were selected to be between 70 and 200 bp with primers designed to contain ≤40 % GC where possible. Primers were purchased as desalting-purified lyophilized material from Sigma-Aldrich (St. Louis, MO) and reconstituted with sterile Tris–EDTA (TE) buffer (10 mM Tris–HCl, 1 mM EDTA Na_2_, pH 8.0, Promega, Madison, WI) to a concentration of 500 µM. Aliquots of each forward and reverse primer were made by dilution to a concentration of 5 µM in TE buffer. All primer stocks were stored frozen at −20 °C until plated for array assembly. Genomic sequence for the guinea pig (*Cavia porcellus*) was downloaded from the Ensembl database (http://useast.ensembl.org/Cavia_porcellus/Info/Index); sequence for MG (prototypic laboratory strain G37) was obtained from GenBank (http://www.ncbi.nlm.nih.gov/genbank/).

### Primer Validation

**Fig. 1:**
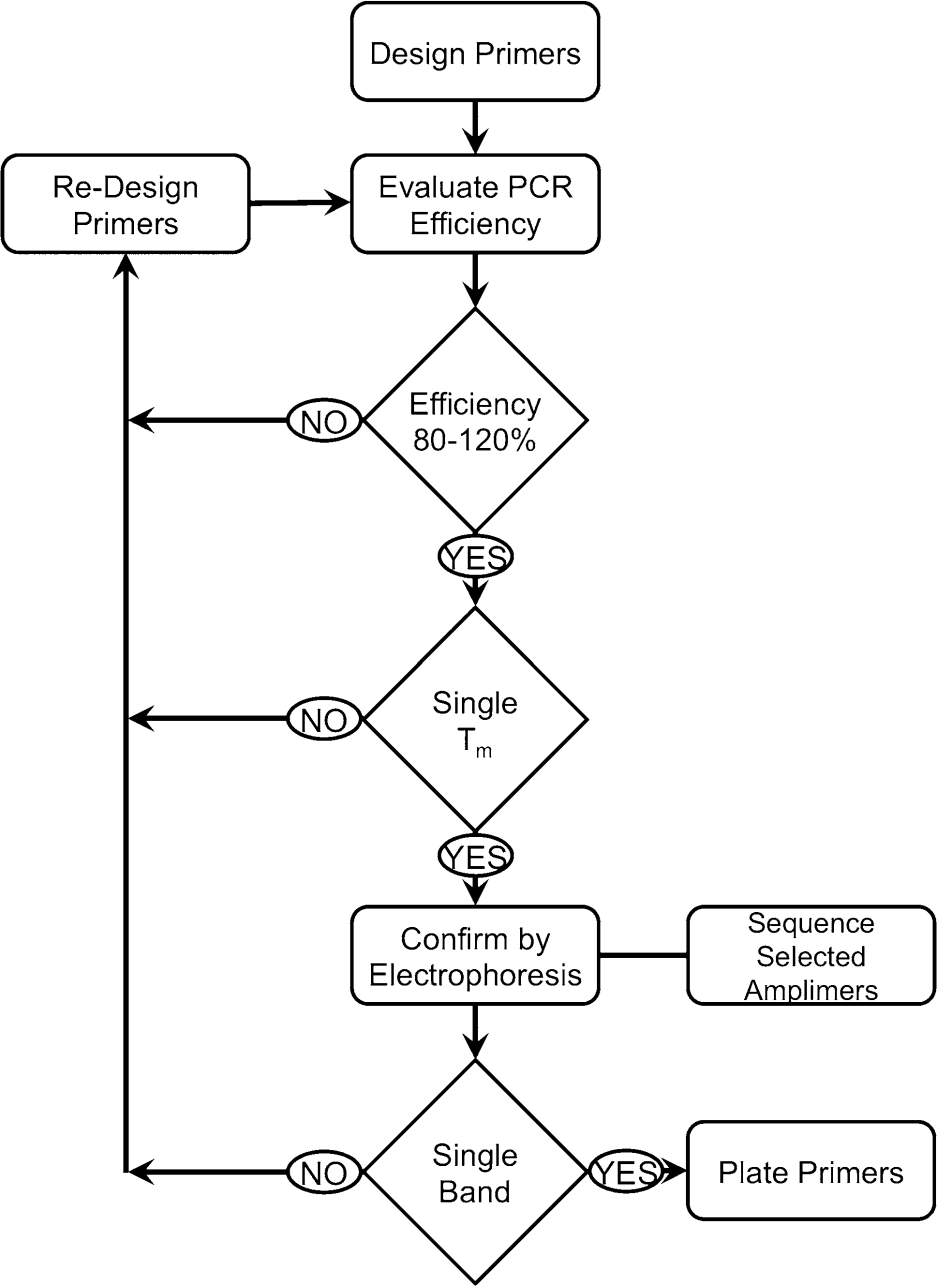
Generalized workflow for primer validation. Primers first met PCR efficiency criteria (80–120 %). Secondary evaluation of target specificity used high resolution melt temperature analysis to confirm the presence of a single melt peak. Final confirmation of primer specificity utilized electrophoresis through agarose to ensure only one product was produced, validating the primer pair, and establishing the expected *T*_m_ for each corresponding amplimer. Approximately 40 % of all amplimers were sequenced with 100 % confirmation of their identities

Primer pair candidates were required to pass two validation criteria before being considered for inclusion onto an array assembly. A generalized flow chart of this primer pair validation process is shown in Fig. 1.

**Fig. 2:**
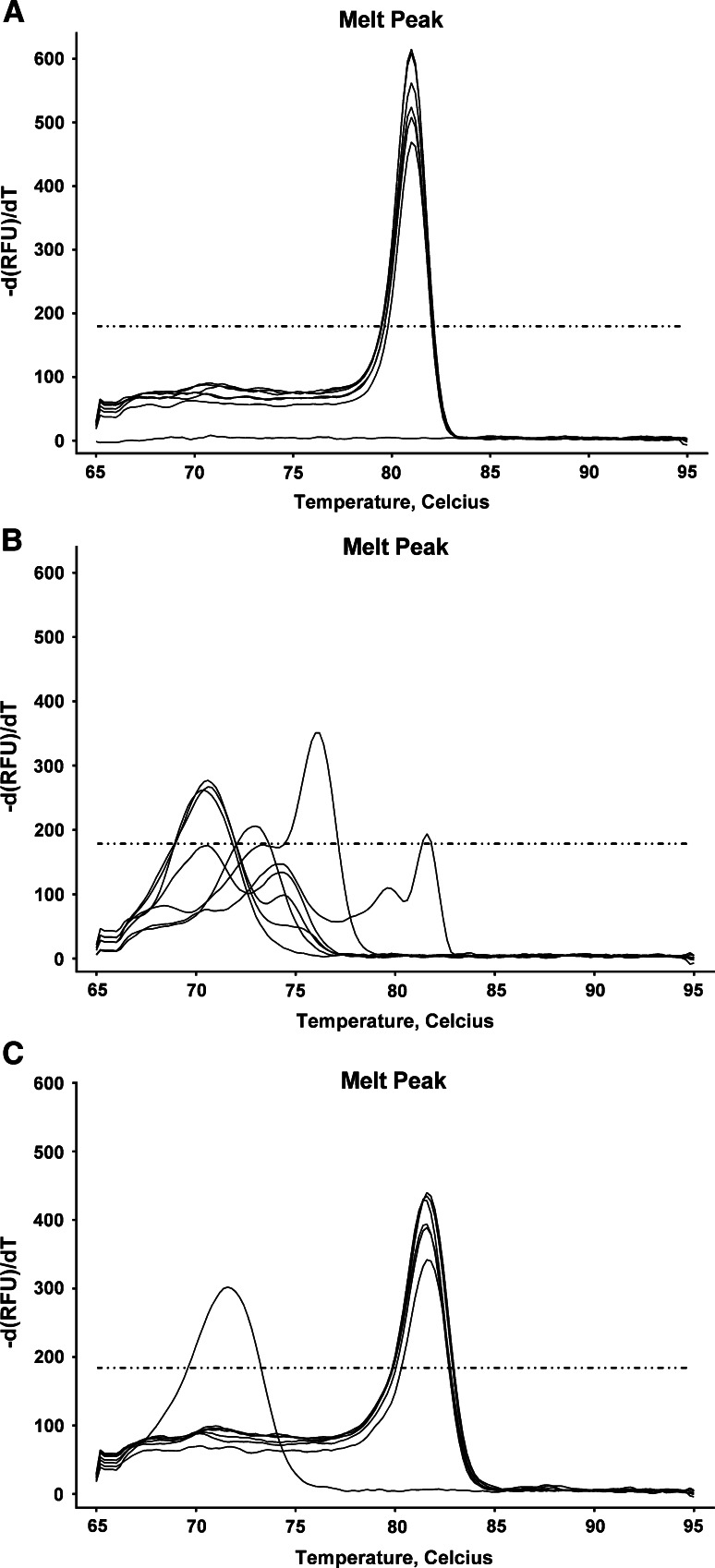
Secondary primer pair validation was conducted by melt temperature (*T*_m_) analysis to confirm target specificity. All reactions were carried out as three, 10-fold dilutions analyzed in duplicate. **a** A *single melt peak* indicated production of one product and a passed secondary evaluation. **b** *Multiple melt peaks* were indicative of off-target primer binding or primer-dimer formation, resulting in multiple amplification products. The lower panel primer pair did not pass validation and was re-designed. **c** An example of a primer pair that produced a primer-dimer in the no template control well. This primer pair passed validation as the *T*_m_ of the primer-dimer was ≥5 °C from the expected *T*_m_. All panels: the *dotted line* indicates the baseline threshold

Equivalent PCR efficiency is an essential aspect to the effective development of a multi-target system to be used on one sample to produce semi-quantitative data. Based upon extensive experience in designing and implementing quantitative PCR (qPCR) assays [13–18], efficiencies between 80 and 120 % with associated correlation coefficients ≥0.95 are required to provide optimal data for comparing transcription levels of selected genes within a sample and to ensure correct normalization across comparator samples. To determine the PCR efficiencies of newly designed primer pairs, a PCR master mix was made (“PCR” section) for each primer pair set and a series of three, and 10-fold dilutions of DNA (for MG primers) or cDNA (for *Cavia porcellus* primers) were prepared and used as the template for the PCR. This series of dilutions served as a calibration curve that provided an accurate assessment of efficiency for each putative gene target. Additionally, a negative template control (NTC) was included on all validation runs to identify primer pairs that formed primer-dimers in reactions with little or no target sequence. Amplification of a primer pair that results in a PCR product from primer-dimer formation may still be considered for inclusion on an array build provided that the *T*_m_ obtained varies by at least 5 °C from the *T*_m_ of the expected product (Fig. 2c). If a primer pair results in a PCR efficiency of 80–120 %, primer pair specificity is next confirmed to ensure the fidelity of the reaction.

Primer specificity was a second and equally important consideration during the validation process. Confirmation of the identity of each amplimer was completed by melt temperature analysis at the end of the amplification cycles to create a specific *T*_m_ for each of the amplified products. A single *T*_m_ suggested that only one PCR product was produced. In cases of single *T*_m_ (Fig. 2a), agarose gel electrophoresis confirmed the product size. Successful amplification was indicated by a single band that corresponded to the predicted amplimer size. During optimization, if multiple *T*_m_s (Fig. 2b) or a band of unexpected size was observed, the primer pair was considered sub-optimal, and was subjected to re-design and re-validation. Sanger sequencing of randomly selected PCR products (to date approximately 40 % of the total targets) showed 100 % specificity and was achieved using our validation approach. The *T*_m_ established for each successful primer pair was used as a subsequent quality control evaluation for experimental runs prior to analyses of gene expression differences. This workflow, using the optimized KS protocol and refined PCR primer pair design parameters, created an overall success rate of 80 % for over 400 first-round primer pairs tested to date. Second round re-design led to success for the majority of the targets, although 5 % required a third round of design, suggesting that a small subset of target genes may fail our array approach.

### Assembly of Array Plates

Validated optimized primer pairs were robotically plated into 96-well skirted PCR plates. Use of a 12-channel pipet for distribution of primers to array plates was confirmed to be a viable, although laborious, alternative to robotic plating. After array plating, multiple array plates were chosen at random and tested to address quality and contamination concerns. Water only control PCR confirmed that no contamination occurred during the plating process. Assembled array plates were tested using target nucleic acid to confirm primer activity conformed to results obtained during optimization and validation studies.

For the guinea pig, host immune response transcription array (gpArray), two additional quality controls were conducted after array plates were produced. Purified Vero and human DNA were tested in the gpArray to determine if the guinea pig primer pairs would recognize homologies in those genomes. These DNAs were specifically selected to identify possible sources of contamination that could be introduced at the time of sampling or during downstream sample manipulation: human DNA from laboratory personnel collecting and preparing the samples, and Vero cell DNA to control for extraneous genetic material present in virus stocks grown in Vero cells and introduced during inoculation of test animals. Interestingly, both Vero and human DNA were recognized by the guinea pig primers (data not shown) and resulted in successful amplification of gene targets. Melt temperature analysis of the corresponding PCR amplimers showed that 82 % of the resulting Vero *T*_m_s and 85 % of the human *T*_m_s were sufficiently different (±0.8 °C) to be distinguished by our standard quality assessment prior to data analysis. Of those targets that were ≤0.8 °C, only 7 Vero and 7 human DNA amplimers produced *T*_m_s that were indistinguishable from their guinea pig counterparts.

### RNA Extraction and cDNA Synthesis

Template RNAs were extracted using the Aurum Total RNA 96 kit (Bio-Rad) following the manufacturer’s instructions and included an on-column DNase I digestion to remove genomic DNA contamination. Prior to reverse transcription of the RNA using the iScript cDNA Synthesis kit (Bio-Rad), lack of DNA contamination was confirmed using a Taqman qPCR targeting the housekeeping gene glyceraldehyde 3-phosphate dehydrogenase (GAPDH). cDNA synthesis reactions were carried out in a final volume of 60 µl per sample and contained the following reaction components: 12 µl 5x iScript Reaction Mix, 3 µl iScript Reverse Transcriptase (225 units), and 45 µl template RNA (~600 ng). cDNA synthesis was carried out using a three-step PCR protocol of 25 °C for 5 min followed by 30 min at 42 °C and a final 5 min incubation at 85 °C to stop the reaction. Resulting cDNAs were stored frozen at −20 °C until assayed.

### Production of Arrays

For array plating, a fresh 1 ml primer stock containing both forward and reverse primers for each gene target was prepared in a sterile 1.8 mL cryovial at a final concentration of 434.8 nM in TE. This concentration of primer stock was used to ensure a final 0.2 µM concentration for both forward and reverse primers upon addition of reaction mix (see next section “PCR”) to all wells of the array plate. The aliquots were stored on ice until primer mixes were produced for all targets on a given array. The cryotubes were briefly centrifuged (300 RCF, ~30 s) before being pipetted into 96-well skirted PCR plates (Thermo Fisher Scientific, Waltham, MA) at a volume of 11.5 µl per well using sterile RNase/DNase-free filter tips (Mettler-Toledo, Columbus, OH). Initial plating was accomplished using a 12-channel pipet (Mettler-Toledo), however subsequent plating utilized a Tecan Evo 200 robotics platform (Tecan US, Inc., Morrisville, NC) to increase the throughput, efficiency, and accuracy of primer distribution. Finished array plates were covered with sterile foil sealing tape (Thermo Fisher Scientific) and centrifuged briefly (300 RCF, ~30 s) to collect the liquid in the bottom of the wells. Array plates were stably archived at −20 °C for long-term storage until used for sample screening. Validated gpArray and MG Array plates can be obtained from our lab group under MTA or through collaboration.

### PCR

All PCR array reactions were completed in 96-well plates in a total volume of 25 µl. Prior to assembling the reaction mix, template cDNA was diluted 1:5 in sterile DNase/RNase-free water. The final volume for the PCR and dilution of starting material is a best practice suggestion based on our experience with PCR development [13–18]. The final reaction volume and/or cDNA dilution may be adjusted as determined by the end user through experimental in-house validation. The reaction mix was assembled using 12.5 µl of 2× iQ SYBR Green Supermix (Bio-Rad) and 1 µl of diluted cDNA for each well to be tested. A volume of 13.5 µl of the SYBR-cDNA reaction mix was then added to each well of an assembled array plate containing primers (0.2 µM final primer concentration) using a 12-channel pipet and low retention, RNase/DNase-free, filtered pipet tips (Mettler-Toledo). Plates were then sealed with optical tape and briefly centrifuged (6000 RCF, ~30 s) to collect the liquid at the bottom of the wells prior to PCR. Reactions were carried out on either CFX or CFX Connect Real-Time PCR detection systems (Bio-Rad) using the optimized KS protocol consisting of an 8 cycle amplification of 95 °C for 30 s, 48 °C for 30 s, and 72 °C for 30 s followed by a 40 cycle amplification of 95 °C for 15 s, 56 °C for 20 s, and 72 °C for 20 s during which real-time data were acquired at the annealing step. *C*_q_ analysis was completed by the Bio-Rad CFX Manager software with a constant baseline adjustment to 50 relative fluorescent units for all array runs to provide for accurate comparisons of *C*_q_ values across gene targets.

A high resolution melt temperature analysis was included in the thermocycling program following the second amplification step. Starting at 65 °C, the temperature was raised incrementally by 0.2 °C every 5 s, followed by data acquisition to an ending temperature of 95 °C. *T*_m_ values for all amplicons were evaluated by comparing each corresponding *T*_m_ to established historical values with an acceptable range of ±0.8 °C prior to data analysis.

Reference genes were needed to ensure reliable normalization across samples. However, the expression of many widely used reference genes can vary due to the tissue or cell type from which the sample is collected [10]. It has been shown that multiple reference genes should be used for normalization to avoid unintentional biasing of the results [19]. Thus, for all array runs, gene expression levels were normalized using the mean of four reference genes included on each plate using ΔΔ*C*_q_ [19, 20]. Fold change was calculated by the normalized gene expression of the test sample divided by the normalized gene expression of the control sample. Values greater than one represented an up-regulation and were considered equal to the fold change. Fold change values less than one were down-regulation and were represented by the negative inverse of the fold change.

### Cloning of PCR Amplimers

Selected PCR amplimers (~40 % of targets) of the proper size created by initial array runs were purified (QIAquick PCR Purification kit; Qiagen, Valencia, CA) and then cloned using the TOPO TA Cloning kit (Life Technologies, Carlsbad, CA). Selected clones that were within 0.2 °C of their corresponding control were sequenced using an ABI Prism 3130XL Genetic Analyzer (Carlsbad, CA). Positive clones were also used to confirm *T*_m_ values and were archived for use in future single target qPCR assays.

### qPCR Validation of Array Results

Each qPCR mix contained 1X iQ SYBR Green Supermix, 0.2 µM of each forward and reverse primer, and 1 µl template cDNA. Cloned amplimers for each gene target were included on each plate as a series of 10-fold dilutions (10^6^–10^2^) as quantitation standards to provide a means of calculating the quantities of each target gene. Two additional qPCRs were conducted in parallel using the housekeeping genes hypoxanthine phosphoribosyltransferase 1 (HPRT1) and beta actin to control for cDNA quality and quantity. Negative template controls were included to ensure PCR integrity and indicate potential contamination. All reaction efficiencies were between 80 and 120 % with correlation coefficients of >0.95. Assay sensitivity for these reactions allowed for 100 copies to be detected 100 % of the time. *T*_m_ analysis was used to confirm primer specificity and amplimer identity as described above. Target gene expression levels were normalized using the geometric mean of the housekeeping genes HPRT1 and beta actin prior to analyses. The mean of normalized test sample values for each target gene were divided by normalized control sample values to indicate the fold change for comparisons to array results. Values greater than one represented an up-regulation and were considered equal to the fold change. Fold change values less than one were down-regulation and were represented by the negative inverse of the fold change.

### Guinea Pig Splenocyte Cell Culture

Female Hartley guinea pigs (250–350 g) were obtained from Charles River Breeding Laboratories (Wilmington, MA) and housed in American Association for the Accreditation of Laboratory Animal Care-certified facilities. Animal studies were approved by the Institutional Animal Care and Use Committee (IACUC) at the University of Texas Medical Branch (UTMB), and all animals were humanely euthanized following UTMB IACUC-approved procedures. Guinea pig spleens were then harvested using sterile technique and placed into Hank’s Balanced Salt Solution (HBSS; Corning Life Sciences-Mediatech, Inc., Manassas, VA) supplemented with 5 % (vol/vol) newborn calf serum (Life Technologies Incorporated, Carlsbad, CA) and 1 % penicillin/streptomycin (10,000 U/ml penicillin/10,000 µg/ml streptomycin stock; Sigma-Aldrich). All manipulations of the tissues were conducted at room temperature. Spleens were minced and pushed through a metal mesh sieve to produce single-cell suspensions as described previously [21]. Cells were pelleted and washed with HBSS a total of 3 times. Erythrocytes were lysed by exposure to acetic acid prior to splenocyte enumeration. Cells (1 × 10^6^ cells/well) were seeded into 24-well plates in medium containing RPMI 1640 (Corning Life Sciences-Mediatech, Inc.), 10 % (vol/vol) fetal bovine serum (FBS; Corning Life Sciences-Mediatech, Inc.), 1 % penicillin/streptomycin, 1 % l-glutamine (Sigma-Aldrich), 1 % sodium pyruvate (Sigma-Aldrich), 1 % non-essential amino acids (Sigma-Aldrich), and 50 µM β-mercaptoethanol (Sigma-Aldrich). Half of the wells were further supplemented with 50 ng/ml phorbol 12-myristate 13-acetate (PMA) and 1 mM ionomycin to stimulate the cells. Cells were incubated (37 °C, 5 % CO_2_) for 24 h and then re-suspended in 0.2 ml Aurum Total RNA Lysis Solution (Bio-Rad, Hercules, CA) containing 1 % BME, vortexed and stored at −80 °C until used for RNA isolation and cDNA preparation.

### Statistical Analysis

Comparisons to detect significant variations in expression for each target gene were carried out using a two-tailed Student’s *t* test after 2^−Δ*C*q^ conversion of individual *C*_q_ data to a linear form [20]. Comparisons between gene expression levels from qPCR assays were made using a two-tailed Student’s *t* test after log conversion of the qPCR results for each target with Prism software (v6.0; GraphPad, La Jolla, CA). For all comparisons, a *P* value of 0.05 was used to designate significance.

## Results

Development of the RT-PCR arrays first required the selection and optimization of a permissive thermocycling protocol that would allow multiple diverse primer pairs of differing annealing temperatures to amplify targets with similar PCR efficiencies. After optimizing a PCR thermocycling protocol, we refined sequence target selection parameters to create an optimal primer design paradigm that increased the success rate for selecting array-compatible primer pairs. Additionally, we established target quality control and validation methodologies to ensure adequate PCR efficiency and to confirm primer specificity. Finally, using optimized methods two disparate example array assemblies were completed and tested to confirm the flexibility and utility of our approach.

### Assembled Transcription Arrays

Several genetically distinct target sets have been successfully evaluated using our optimized array design and assembly methods. As proof of concept, we offer results for two distinct types of biological target sets with disparate goals and outcomes.

*MG Array* Our initial efforts to develop an array platform were driven by our research into the under-studied bacterial pathogen MG [16, 22]. This bacterium is responsible for genital tract infection and can develop an intracellular niche leading to persistent colonization of an infected individual [23]. MG provides a unique opportunity for studying the genes associated with infection and the establishment of persistence as it contains one of the smallest genomes capable of self-replication. To better understand the pathogenesis of this bacterium, we designed primers to target 188 conserved genes representing ~40 % of the known genes of this organism. Using the optimized workflow, the success rate for first-round primer design was 83 % with an 80 % success rate for the re-design of suboptimal primer pairs.

**Fig. 3:**
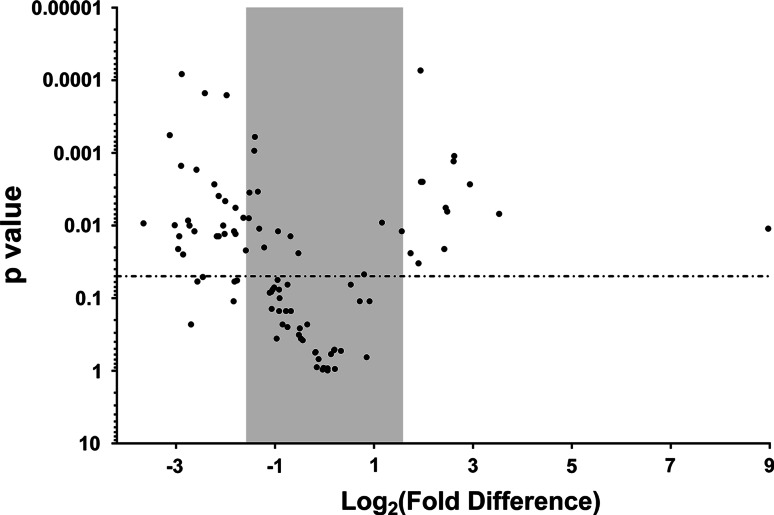
Comparisons between MG 2300 and MG 2341 showed significant transcription level changes in 42 % of genes evaluated. A plot comparing MG 2300 to MG 2341 showed that significant up-regulation was detected for 13 genes (*upper right quadrant*). Additionally, 27 genes were significantly down-regulated (*upper left quadrant*). The gray rectangle indicates a 3-fold up- or down-regulation. Approximately, 58 % of observed gene transcription levels were found to fall within this range and were considered unchanged. Selected genes were evaluated by qPCR, and both their magnitude and direction of change were in agreement with the data obtained from the array. The *dotted line* indicates a *P* value of 0.05

The assembled two 96-well plated array (Supplementary Table S1) was utilized to study transcription profiles of two similarly cultured clinical isolates, MG 2300 and MG 2341 [24], under normal growth conditions. Array profiling of these two cultures showed 13 genes that were significantly up-regulated in MG 2300 compared to MG 2341 under similar conditions (Fig. 3). Additionally, 27 significantly down-regulated genes were identified in the MG 2300 culture compared to that of cultures containing isolate MG 2341.

*Guinea pig array (gpArray)* Due to its similarity to humans, the guinea pig is often used to study human infection and disease but currently suffers from a dearth of associated immunologic reagents [25, 26]. To increase our understanding of the immune response in these animals, we utilized the flexibility of our platform to develop an array capable of characterizing transcription changes in the guinea pig. First-round primer design success rates for the gpArray were 73 % with an associated 50 % success rate for primer re-design, consistent with the greater complexity of this genome compared to that of MG. Supplementary Table S2 provides a summary of primer pair sequences and associated *T*_m_s for the gpArray. Final array assessment was conducted using guinea pig splenic cells cultured with phorbol myristate acetate (PMA) plus ionomycin, an immunostimulatory cocktail known to activate lymphocyte proliferation and cytokine production. These samples were studied for known shifts in transcription levels associated with PMA/ionomycin stimulation.

**Fig. 4:**
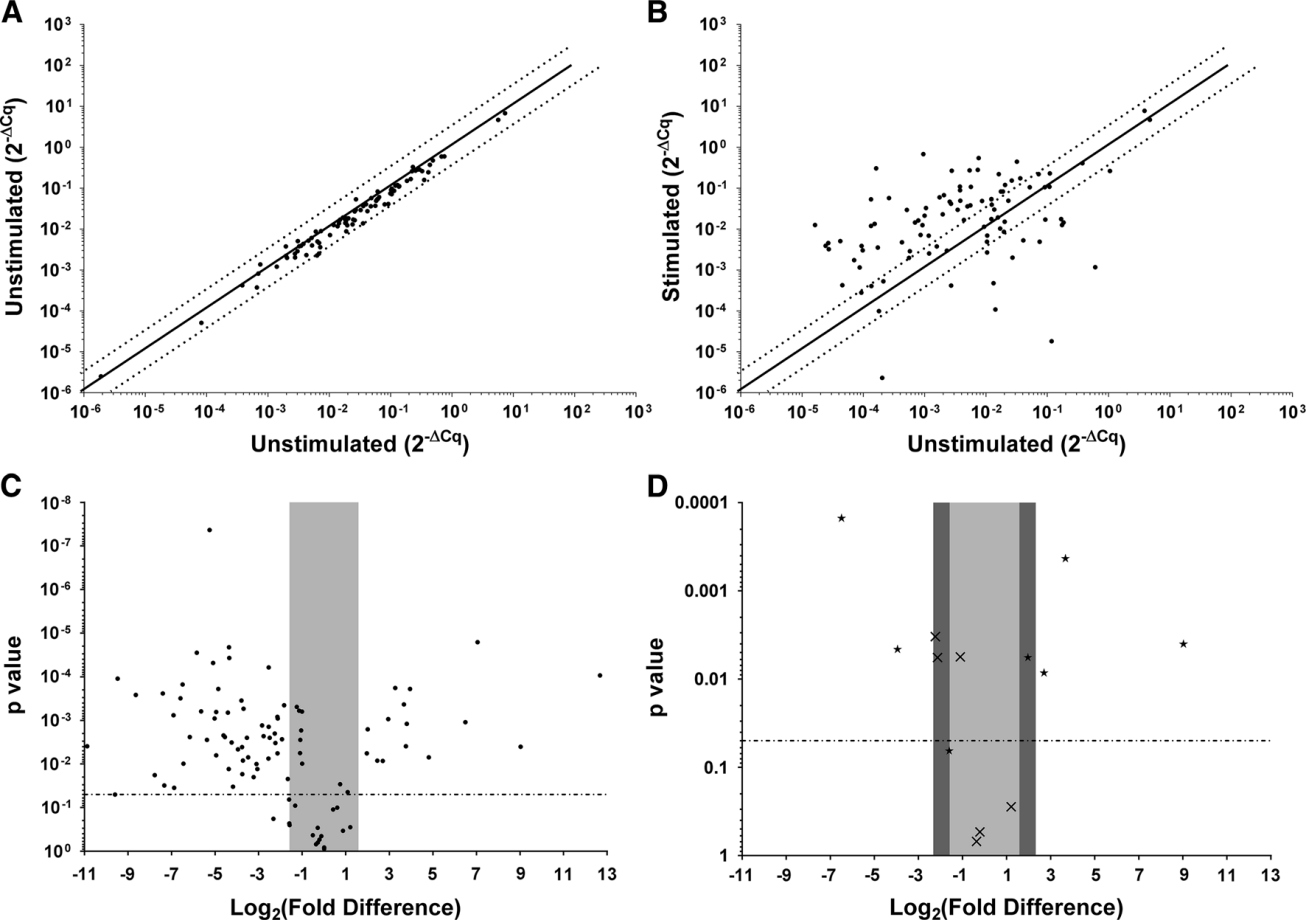
Initial validation of the gpArray using guinea pig splenocytes treated with medium only for 24 h (unstimulated) or PMA/ionomycin (stimulated). Both (**a**) and (**b**). The *solid line* indicates baseline expression; the dotted lines indicated 3-fold up or down expression differences. **a** Replicates of unstimulated splenocytes showed no differences validating the reproducibility of the array and establishing the level of biological and technical noise. **b** When stimulated splenocytes were compared to unstimulated samples, 16 % of genes were found to be up-regulated, 60 % were down-regulated, and the remaining genes showed no change. Both (**c**) and (**d**). The *dotted line* indicates *P* value of 0.05. The light gray rectangle indicates a 3-fold up or down change in expression levels. Gene transcription levels within this range were considered unchanged. **c** Statistical significance of expression level differences from stimulated splenocytes compared to unstimulated; 15 genes were significantly up-regulated (*upper right quadrant*) and 55 were significantly down-regulated (*upper left quadrant*). **d** qPCR results were concordant for direction of change and *P* value for 54 % of genes evaluated (indicated by *Black filled star*). Genes that were discordant with respect to direction, *P* value or both were found within a 5-fold up- or down-regulation and are denoted by (×). The dark *gray rectangles* indicate ±5-fold change in expression levels with all genes that showed >5-fold change corresponding to 100 % concordance with array data as confirmed by qPCR

We first compared gpArray data from 2 sets of duplicate cultures of unstimulated splenocytes (1 × 10^6^ cells/sample). A scatter plot (Fig. 4a) of this comparison showed that no genes were up- or down-regulated more than 3-fold, indicating that there were no meaningful differences in transcription between these unstimulated samples. This method also helped to establish the level of biologic and technical noise in the PCR array. Comparison of array data from stimulated splenocytes with the untreated control values identified a number of genes that were differentially expressed (Fig. 4b). When these results were statistically analyzed, transcription levels of 15 genes were significantly up-regulated and the transcription levels of an additional 55 genes were significantly down-regulated (Fig. 4c).

### Confirmation of Identified Differential Gene Expression

Sampling error and limits of detection can be serious confounders for generalized array approaches. To address these concerns, we utilized subsequent qPCR that included 10-fold dilution series of cloned amplimers from the gpArray as standard curves. Single target qPCR allowed for larger sample size with increased statistical power to confirm array, indicated shifts in the transcriptome of the stimulated cell population. To establish this method and effectively define confidence in meaningful array-based differences, we selected qPCR targets representing the highest up- and down-regulated differences as well as differences representing mid- and minimally different fold changes established in the arrays. Most of these targets were deemed as significantly different in treated guinea pig splenocytes.

Initial evaluation of the array results suggested ±3-fold change would likely be meaningful but in pilot studies that were intentionally underpowered, we discovered ±5-fold to be more accurate. Comparison of the array and qPCR data (Table 1) confirmed that all fold changes >5-fold were concordant and significant comparing triplicate stimulated samples to unstimulated controls (*P* < 0.05). Of eight targets tested by qPCR with <5-fold difference indicated by the array, 2 matched both direction and *P* value, 3 matched direction only, and the remaining 3 were discordant for both direction and significance (Fig. 4d**).** gpArray results were found to be ≤5-fold change and therefore should be confirmed by an alternative method. Importantly, all values of >5-fold change are expected to be solidly concordant.

**Table 1:** qPCR validation of expression level changes from PMA/ionomycin-stimulated guinea pig splenocytes compared to unstimulated cells

Gene	Array indicated fold change	Array *P* value <0.05	qPCR fold change	qPCR *P* value <0.05
IFNγ	522.5 ↑	Y	2.3 ↑	Y
TLR-3	89.9 ↓	Y	1.4 ↓	Y
CD8α	15.4 ↓	Y	1.1 ↓	Y
CXCL10	12.8 ↑	Y	1.6 ↑	Y
OX40L	6.5 ↑	Y	4.7 ↑	Y
CD107b	4.7 ↓	Y	1.0 ↓	N
CD107a	4.4 ↓	Y	1.0 ↓	N
IL-21	3.9 ↑	Y	1.8 ↑	Y
B2 M	3.0 ↓	N	1.0 ↓	N
IFNAR1	2.3 ↑	N	1.2 ↑	Y
CXCR3	2.1 ↓	Y	1.3 ↑	Y
TGFβ	1.3 ↓	N	1.2 ↑	Y
CD28	1.2 ↓	N	1.0 ↑	N

Y: Yes, indicates a *P* value <0.05; N: No, indicates a *P* value >0.05; ↑: indicates up-regulation; ↓: indicates down-regulation

## Discussion

The work described here illustrates the utility of our array platform by providing two new resources for the evaluation of transcriptional profiling for organisms that currently suffer from a lack of commercially available assays. By utilizing the reliability of SYBR green-based chemistries [27], we have developed a cost efficient, highly specific, and reproducible methodology that can be adapted to any target species for which whole or partial annotation of its genome is available. The costs for the development of an array are based upon several of the parameters we detail in our method. Specifically, we estimated, on average, each primer pair evaluation, including the cost of the primers and PCR mix, was $32USD. Given the reported success rate of designed primers, the cost of development and validation of 96 compatible gene targets was under $4,000USD. Once established, a 96 target array costs $125USD/sample.

Our enhanced array system builds on previous primer design and PCR parameters [12] to create a system compatible with standard molecular biology laboratories. Our array platform can accommodate any number of genes spanning multiple 96-well plates, making it feasible to screen one sample across many genes or narrowing the focus to evaluate only a few important genes using a “mini array.” In this manner, multiple samples may be assayed per plate, reducing costs while increasing throughput. Thus, the platform is an ideal screening tool for detecting transcription changes from individual or biologically pooled samples to identify relevant differentially expressed genes for subsequent evaluations. Additionally, the targets included on each array can be converted easily to specific sensitive qPCR assays using the primer sets already incorporated on the platform. Such assays enable efficient confirmation of the transcriptional changes detected by an array for many individual samples. Finally, our approach allows for the construction of a secondary array using only those genes identified and confirmed by qPCRs, further reducing overall costs and increasing the efficiency of array screens.

There has been intense development in the area of transcriptomics and associated technologies in recent years [5], resulting in several options for gene expression analysis. RNA-seq can provide de novo evaluation of gene expression in the absence of a reference genome [5], making this an attractive option for researchers working with an uncommon research species. However, this technology can be cost-prohibitive (~$250–3,000USD/sample) and requires a multi-step sample preparation that can introduce unwanted bias into the results [7, 8]. In contrast, PCR arrays are relatively inexpensive on a per sample basis and can provide a more user-friendly approach to transcription analysis. Unfortunately, while many signaling pathways and disease states may be interrogated using commercially available PCR arrays, the limited number of target species prevents study of many important animal models. Custom arrays may be requested, however there is an associated lag time for their development that, coupled with increased costs, lessen their appeal. The optimized platform we have developed addresses these issues. Additionally, our methodology costs only $125USD/sample and provides an alternative to other transcription assessments, making it a more cost-effective and reproducible means of approaching expression analysis for non-commercially available species or signaling pathways. The array method also provides for the opportunity to combine targets from host and pathogen assemblies to coincidentally evaluate two target species in one assay further enhancing applicability.

Initial development of the array platform was driven by our research into the emergence of MG as a sexually transmitted pathogen [28, 29]. With the availability of commercial reagents lacking for such an under-studied organism, our goal was to provide an important tool for understanding the transcriptome of this bacterium. Thus, the MG array was developed to include individual genes that are crucial to motility [30, 31], important in regulating responses to environmental changes [32], and other potential pathogenicity cues [33].

Utilizing the MG array, we investigated the variability in gene transcription between different clinical isolates to identify genes important to survival or adaptation to the host environment. Screening detected transcriptional changes that could be associated with drug resistance or immune evasion. These observations resulted in a more refined list of gene targets and the flexibility of our platform provided the ability to easily re-configure a more specific focused version of the MG array. This refined array will be used to identify new targets for therapeutic interventions and vaccine development.

As evidence of success with a more complex genome, we developed an array for the guinea pig because it is an excellent model for the study of infectious diseases [26, 34–37]. The guinea pig boasts immune and pathophysiologic similarities to humans that make it a preferred animal for studying disease pathogenesis [38–40] and for evaluating new vaccines [41, 42]. Despite the value of this small animal model, full utilization of possible outcomes has been hampered by a lack of commercially available immunological assays. Independent researchers have attempted to overcome these limitations, however the resulting assays are often specific for only a single target [25, 43, 44], restricting their utility to a smaller subset of research applications.

Using the validated gpArray, pilot studies were completed to compare PMA/ionomycin-stimulated splenocytes to unstimulated cells to identify changes in expression levels associated with this non-specific immune activator cocktail. This approach also helped to validate the sensitivity of the gpArray providing defined cutoffs for meaningful differences identified by the array. As expected, the expression levels of interferon gamma (IFNγ) increased significantly compared to unstimulated controls. Not surprisingly, additional interferon-stimulated genes (CXCL10 and CXCL11) were also found to be up-regulated along with several interferon-induced lymphocyte activation markers (CD69 and CD223). These results were in agreement with known lymphocyte activation [45–47] and confirmed that the gpArray could detect transcriptional changes and could be useful as a screening tool.

The changes in expression levels that were observed were confirmed by subsequent single target qPCR to validate initial array results. The qPCR of selected targets evaluated both large and small fold changes in up- and down-regulated genes, and showed that transcription level changes of ±3-fold were less meaningful than suggested by initial array evaluations. Importantly, qPCR evaluations showed 100 % concordance for expression changes of >5-fold and indicated that changes <5-fold should be confirmed by alternate approaches or increased sample size. Single target qPCR adapted from the existing primers on the array proved an efficient and straightforward means of addressing both statistical confirmation and increased numbers of samples analyzed in cost-effective fashion.

Collectively, the presented data showed that our array development platform was a reproducible and highly adaptable resource for examining changes at the transcriptional level in disparate organisms that lack optimal assays for research. Importantly, the system can be used with only partial genomic sequence from pathogens and commensal organisms that are not yet cultured in a laboratory as well as from more complex species currently lacking a fully annotated genome. To our knowledge, the MG array created by our method represents the first resource for evaluating large-scale expression level changes in this emerging, sexually transmitted pathogen. Additionally, while microarray technologies have been used to study the guinea pig previously [48, 49], restrictions inherent to these technologies (e.g., increased costs and diminished throughput that can reduce statistical power) can be overcome using RT-PCR arrays. With real-time PCR instruments increasingly common in research laboratories, we believe RT-PCR arrays to be more approachable and easily adopted by scientists studying emerging organisms or species lacking available assays. In addition, this developmental platform has the potential to create tools to study multiple organisms present in a single sample, i.e., pathogen in the context of host, providing a simple and accurate new screening tool for gene expression analysis.

## Electronic supplementary material

Below is the link to the electronic supplementary material.

Supplementary material 1 (DOC 523 kb)

Supplementary material 2 (DOC 280 kb)
