# Supplementary material for: Development and Utilization of a Custom PCR Array Workflow: Analysis of Gene Expression in Mycoplasma genitalium and Guinea Pig (Cavia porcellus)
Source: Mol Biotechnol. 2014 Oct 31;57(2):172–83. doi: 10.1007/s12033-014-9813-6 (PMC4298676; doi:10.1007/s12033-014-9813-6)
Supplement: Supplementary file 1 — Supplementary material 1 (DOC 523 kb) [file 12033_2014_9813_MOESM1_ESM.doc]

**Supplementary Table S1** List of primers included on the MG array

| **Gene Description** | **MG Primer Reference #** |  | **Sequence (5' - 3')** | **Length (bp)** | **Amplimer T_m_ (°C)** |
| --- | --- | --- | --- | --- | --- |
| hypothetical protein MG_011 | 11 | F | TCGTCAGCACTATTAACTA | 112 | 76.0 |
|  |  | R | CTGTCAATTCTTCTCTTCTTA |  |  |
| chaperone protein DnaJ | 19 | F | TTCCCTATAACCTTGATATTG | 170 | 78.5 |
|  |  | R | GCCATTACAATCCTTACAA |  |  |
| elongation factor P | 26 | F | ATGGCAGAAATGATAGAAG | 103 | 76.4 |
|  |  | R | CGGTTTTGTTAAATGTATTTTC |  |  |
| hypothetical protein MG_028 | 28 | F | GGAGTAGTTTTACTTGTTGTA | 113 | 73.5 |
|  |  | R | GTTCCTGTTGTTGATCATA |  |  |
| uracil phosphoribosyltransferase | 30 | F | CTCTGATTCACATGTTATTATTC | 183 | 76.4 |
|  |  | R | GCTGCAAGAAATATATCAAC |  |  |
| thymidine kinase | 34 | F | TGGGTAAATATCAACCATC | 107 | 77.0 |
|  |  | R | CCGTTTTATTTTGTGAAGTA |  |  |
| putative lipoprotein | 40 | F | TGCCAAACTCTTAACAAA | 99 | 75.2 |
|  |  | R | TCACCATCATTATCTGTTG |  |  |
| phosphocarrier protein HPr | 41 | F | CAAGCAGTTATTAAAGACC | 97 | 78.7 |
|  |  | R | CTACCAGTTTAAGTTCAGA |  |  |
| spermidine/putrescine ABC transporter, ATP-binding protein, putative | 42 | F | GTTGGTAATCCTGTTGATA | 86 | 74.7 |
|  |  | R | GTACCTTTAAAGATGTTAGC |  |  |
| purine nucleoside phosphorylase | 49 | F | GAGGTTGAAACTATCATTAGA | 170 | 77.8 |
|  |  | R | TCCACTAAAGAACTTGTTG |  |  |
| MFS family transporter | 61 | F | CACCATCTTTAATCCAACA | 95 | 75.5 |
|  |  | R | AGGGGTAATTGTAAGTATTG |  |  |
| PTS system, fructose-specific IIABC component | 62 | F | AGGCTGTTATCAATGATAA | 97 | 75.2 |
|  |  | R | TGAGGTAAAGCTAAACTAGTA |  |  |
| ABC transporter, permease protein, putative | 64 | F | GATCCTAGTAGTTATGAAACAA | 94 | 74.8 |
|  |  | R | CTCAGTTGTTAATATCAGGATA |  |  |
| transketolase | 66 | F | CCAACTTGTATTGCTTAAATG | 173 | 77.5 |
|  |  | R | ACCCATCACTTTAATACCA |  |  |
| putative lipoprotein | 67 | F | CCTAGTTTAAGAAATACTGTTG | 200 | 76.1 |
|  |  | R | CTAGCAATAGTTAATGTATTAAGA |  |  |
| 116 kDa surface antigen | 75 | F | GTGCTAATAAACTCAATTTAAC | 166 | 77.3 |
|  |  | R | GTTGCTTTAACTGAATAGG |  |  |
| hypothetical protein MG_076 | 76 | F | CTACCTAACTTAAGATATCTGTTA | 154 | 76.8 |
|  |  | R | CAACCAATTTTACAGTGG |  |  |
| oligopeptide ABC transporter, permease protein (OppC) | 78 | F | TTGGGATTGATATTTGAACC | 86 | 77.7 |
|  |  | R | CACCATTGCTATAATTGCTA |  |  |
| oligopeptide ABC transporter, ATP-binding protein | 79 | F | GCAGTTCATTGGTATTAATG | 128 | 79.6 |
|  |  | R | CAGCAATAATTAAATCAGGTTC |  |  |
| prolipoprotein diacylglyceryl transferase | 86 | F | GCACAATTGGTTTTAGTTA | 101 | 75.8 |
|  |  | R | AGTGGTTATTGAAGTTTGAA |  |  |
| putative lipoprotein | 95 | F | GCACAAACAACTATTAGTAC | 128 | 78.2 |
|  |  | R | TCTCCTAACTTAATCATAGTAAG |  |  |
| uracil-DNA glycosylase | 97 | F | GCCAATTGATACAAAGGTA | 198 | 79.5 |
|  |  | R | TCTGCTCAATTCAATAGGA |  |  |
| hypothetical protein MG_101 | 101 | F | GAAGGTGAAATATGCTAAAC | 84 | 76.0 |
|  |  | R | CTGTCAATATTGGCAAAA |  |  |
| thioredoxin-disulfide reductase | 102 | F | GGCATTGAAAAGGAAGATTA | 89 | 76.0 |
|  |  | R | CCAACTGTTTTACCTTTGTA |  |  |
| ribonuclease R | 104 | F | GTTGGTGAGTTGAACATAA | 140 | 76.2 |
|  |  | R | TTGCCTAGTGTTCTTAATAAG |  |  |
| peptide deformylase | 106 | F | GCACAAGAATATGACATTATTC | 79 | 75.6 |
|  |  | R | GGATGTAAAACAGTTGTTTTC |  |  |
| serine/threonine protein kinase, putative | 109 | F | GCACAGTTAGTTATATGATTC | 164 | 77.3 |
|  |  | R | GGGGTTAATTGACTTGTTA |  |  |
| hypothetical protein MG_116 | 116-v2 | F | GTGTGAAAATTTTCTTTCAGA | 147 | 76.7 |
|  |  | R | TCAGCATTACAAAATATAAGG |  |  |
| ABC transporter, permease protein | 121 | F | AGCACAATTAGAAAGTTGG | 107 | 78.5 |
|  |  | R | CACCATTAATAGCAATATTAATGA |  |  |
| hypothetical protein MG_123 | 123 | F | TGCTGCTGAATTTAAACA | 81 | 73.4 |
|  |  | R | ACCCAACAATTATTAGAAAGA |  |  |
| inorganic polyphosphate/ATP-NAD kinase | 128 | F | ACCTGTTCTCAATAAGTTAAG | 83 | 75.4 |
|  |  | R | CACCTAAAACAAACACATATTC |  |  |
| hypothetical protein MG_129 | 129-v2 | F | TGGTGGTAAGGAAAATTTTG | 153 | 75.8 |
|  |  | R | CCAAGTACAAAACGAAATTG |  |  |
| hypothetical protein MG_131 | 131 | F | GATGCAATACAGTGCTTTA | 198 | 76.2 |
|  |  | R | ATCGCTAAACTTAACAACC |  |  |
| HIT domain-containing protein | 132 | F | CTGGTTTAAACTATGTTTCTAA | 150 | 77.8 |
|  |  | R | GGTAGTTTTCTTCTAAGGAA |  |  |
| hypothetical protein MG_133 | 133 | F | GGTTCTTTTATTAATGACTGG | 119 | 74.8 |
|  |  | R | GGCGGTTTGATAAATTTATAAG |  |  |
| hypothetical protein MG_135 | 135 | F | GCTCTAATTTACTTTAGTTATGTAG | 149 | 77.6 |
|  |  | R | GGATCACTATTGCTTAAGTTAAG |  |  |
| metallo-beta-lactamase superfamily protein | 139 | F | GTACCATACCTTTTAAAGCA | 138 | 77.8 |
|  |  | R | CTCACTAAAATCATCAAAAGTAA |  |  |
| hypothetical protein MG_140 | 140 | F | GAGCAATTCCTATCTTATTTAG | 142 | 78.6 |
|  |  | R | ACAGCAGCTTCTATATTG |  |  |
| transcription elongation factor NusA | 141 | F | TGGTGAGATTATTGACATTG | 140 | 77.2 |
|  |  | R | CAGGTACAACAACTTCAATA |  |  |
| translation initiation factor IF-2 | 142 | F | GTTGCTAACATTATCAAACAC | 83 | 73.8 |
|  |  | R | TCTGCTAACTGTTCATTTG |  |  |
| ribosome-binding factor A | 143 | F | CGCACGATTATTAATGAGA | 113 | 76.4 |
|  |  | R | CGATCATAACAATCAAGATAAAC |  |  |
| hypothetical protein MG_146 | 146 | F | GCACATTTAGCAGTTGTTA | 115 | 75.4 |
|  |  | R | GTTGGATATCATCATGTTCA |  |  |
| putative lipoprotein | 149 | F | GGTGGTAATCTAACTAAACATA | 130 | 77.8 |
|  |  | R | TGCCATTAGTAAAAGATAGTG |  |  |
| adenylate kinase | 171 | F | TGCAGCAATTATCAGTCA | 76 | 76.3 |
|  |  | R | GGTAGTGATATATTCATAAACAA |  |  |
| metal ion ABC transporter, permease protein | 181 | F | TCCAGGGTTTTATCTTACTA | 189 | 77.0 |
|  |  | R | TCAGCATTGTTAGCTTTATAG |  |  |
| oligoendopeptidase F | 183 | F | GGGATCTATCAGTTTTATTAAAC | 200 | 77.0 |
|  |  | R | CTGCTTGTTATAAAGGTAGTTA |  |  |
| adenine-specific DNA modification methylase | 184 | F | AGCAGTTTGAATTAGTTGA | 89 | 74.9 |
|  |  | R | ACCCTGATTTAATTTCACAA |  |  |
| putative lipoprotein | 185 | F | ATGGCAAGATTAAACTAGC | 111 | 77.0 |
|  |  | R | GTTCCACTGGATAATCATTA |  |  |
| nuclease-like protein | 186 | F | GGAAGTTAGCTTTGCAAA | 89 | 77.2 |
|  |  | R | TGCGTTGTATAGATAAAACTG |  |  |
| ABC transporter, ATP-binding protein | 187 | F | GCGATTACCAAGATTTTAAC | 112 | 78.8 |
|  |  | R | CATCCATTAACAATAGTTTAGG |  |  |
| ABC transporter, permease protein | 189 | F | GTGCTTGAAATTCCTATTTATG | 147 | 78.8 |
|  |  | R | CACTCATCTTGAGATTTAACA |  |  |
| DHH family phosphoesterase | 190-v3 | F | TGCCATTAAACAATTTGATAAG | 178 | 79.1 |
|  |  | R | GTTGGTAAATGTCATGTTGATA |  |  |
| MgPa adhesin | 191-v3 | F | CAGCTAACTTTGTTAATGAAA | 110 | 76.2 |
|  |  | R | CTCCTTACCATAGAAAATCTTA |  |  |
| P110 protein | 192-v3 | F | CATCGTTTGTTAATGTCTTTA | 86 | 75.7 |
|  |  | R | ACCAGTTTGGGTAATAATC |  |  |
| DnaJ domain-containing protein | 200-v3 | F | CAAGCTGAATATGAACTG | 74 | 74.0 |
|  |  | R | TTGGGGATAACTAGGTTA |  |  |
| heat-inducible transcription repressor | 205 | F | CGCTTAAGAAGTGTTATTC | 95 | 76.6 |
|  |  | R | ACTGGTAGGTTAATGATC |  |  |
| hypothetical protein MG_211 | 211 | F | CCAGAAACTTATTACTAGTGAA | 115 | 77.0 |
|  |  | R | CTTGGTTAGTTGATCTAAGAA |  |  |
| 6-phosphofructokinase | 215 | F | TCGCTTATCTAACCAAATATG | 119 | 76.0 |
|  |  | R | GTCAGTTAAATCAAGTTCCTTA |  |  |
| pyruvate kinase | 216 | F | ATCCCTGTTTCTATTATGTTAG | 198 | 78.8 |
|  |  | R | CTGACCTATATTCACATCATTA |  |  |
| proline-rich P65 protein | 217-v3 | F | TTCGCTTAAACTCACTTAA | 198 | 77.6 |
|  |  | R | TCTCCAATTCATTGATCTTA |  |  |
| HMW2 cytadherence accessory protein | 218-v3 | F | CACCAAGAAAGTTAACCAA | 146 | 77.3 |
|  |  | R | CAGGTTGAAATTGTTGTTG |  |  |
| hypothetical protein MG_219 | 219-v2 | F | ACCCATAATGAATAGTGATAGTG | 93 | 77.4 |
|  |  | R | GCAGTAGTTGTTAAACTAAGTTC |  |  |
| hypothetical protein MG_220 | 220-v2 | F | AGGACTTATTATGATAGTTAATGC | 76 | 75.6 |
|  |  | R | AGCTGTGGTACTTTGAAA |  |  |
| cell division protein MraZ | 221-v3 | F | GATGCTAAGTTAGATAAAGAGA | 89 | 75.1 |
|  |  | R | GCTAGATATTGTTCATATTGC |  |  |
| hypothetical protein MG_223 | 223 | F | ACCACTTTATTGATATTCCAG | 139 | 77.6 |
|  |  | R | GCCTTAATCTCTTTGTAACTA |  |  |
| cell division protein FtsZ | 224 | F | GGATGAAAATGAAACTCAATTC | 132 | 77.4 |
|  |  | R | CTTGCTAAATTAGGATAGTGATAA |  |  |
| dihydrofolate reductase | 228 | F | CTACGTTATTTCAAGCTTTG | 175 | 76.9 |
|  |  | R | GCTTGGTAACTTTTAATTAG |  |  |
| hypothetical protein MG_237 | 237 | F | CTCCATCCTAATTTAGAAGTAA | 143 | 76.0 |
|  |  | R | GCTCTATCTAACTGTTCATAA |  |  |
| trigger factor | 238 | F | CAGTCGATTAATAAAGCTGAA | 125 | 76.4 |
|  |  | R | GTAGGATAATCATCAATGACATTA |  |  |
| putative lipoprotein | 309 | F | TTTACACCGAGTAAAATGACCATT |  | 79.6 |
|  |  | R | AGATTTTCAATGGTTCTGGAGTTT |  |  |
| P32 adhesin | 318 | F | CCTGATTTATCCCTACAGTA | 93 | 77.3 |
|  |  | R | TGCGCTTTACAATAGGTA |  |  |
| glycerophosphoryl diester phosphodiesterase family protein | 385 | F | GAGACGAATTAATGAGATATTG | 151 | 77.6 |
|  |  | R | AAGCGATCGTTTTCATAA |  |  |
| chaperonin GroEL | 392 | F | TCAGGTTCTAAAGAAATTGG | 115 | 77.4 |
|  |  | R | CAGTGGTTTCTAATGTTGTA |  |  |
| co-chaperonin GroES | 393-v2 | F | TCCCTTTATTAGCATTAGCA | 91 | 75.4 |
|  |  | R | GTGGAATCAAACAAAGAAGAA |  |  |
| F0F1 ATP synthase subunit alpha | 401 | F | CCTTGAACAAAATACAGTC | 159 | 79.8 |
|  |  | R | CTACCATCAATTGCTTCA |  |  |
| F0F1 ATP synthase subunit A | 405 | F | CCTACTGATCAAATTCTTG | 200 | 75.3 |
|  |  | R | TAGGGAGCAAATTTCTTA |  |  |
| phosphopyruvate hydratase | 407 | F | GGATCATTATCACGTAATTCA | 189 | 79.9 |
|  |  | R | GGGAAGTTCAAATCTAAACA |  |  |
| phosphate ABC transporter, ATP-binding protein | 410 | F | GTGAGATGAAGTGAAAGATAA | 102 | 78.6 |
|  |  | R | TCTGGTTGTAAAGCAATAG |  |  |
| LuxR regulatory protein, putative | 428 | F | GAAGCGTTTTAATCCTGAA | 113 | 75.4 |
|  |  | R | CAAGCAATTCAACTTTATTCA |  |  |
| phosphoglyceromutase | 430 | F | TCCCGAAATGTCATGTAA | 144 | 76.8 |
|  |  | R | AGTGCTTCAAGAGCTTTA |  |  |
| triosephosphate isomerase | 431 | F | AGTGGTTCATATACTGGAA | 107 | 75.4 |
|  |  | R | CTGGTTTCGTTATAGTATTTTC |  |  |
| uridylate kinase | 434 | F | CACAGCAAACTATTGATAAAG | 109 | 77.3 |
|  |  | R | ACTGCTCTTAAAGCTAATG |  |  |
| ribosome recycling factor | 435 | F | TGCCTTTAATATCTTTAGCTC | 187 | 77.6 |
|  |  | R | CGAGTTTCTTGAGTAATTTG |  |  |
| type I restriction modification DNA specificity domain-containing protein | 438-v2 | F | ACTCCAAAACTAAAGCTAAA | 138 | 75.4 |
|  |  | R | ACCACCGTTAAAATATTCA |  |  |
| UTP-glucose-1-phosphate uridylyltransferase | 453 | F | GAGCATAAAGAGATTGAAGA | 183 | 77.4 |
|  |  | R | CAAGCATTGTTTTAAAGCAG |  |  |
| ribonuclease P protein component | 465 | F | AGGGTTGCAATATCAATTG | 141 | 76.0 |
|  |  | R | GCCTTTGTTGACAATAACTA |  |  |
| ABC transporter, ATP-binding protein | 467 | F | TGCACTTGATAGTAATTCTG | 141 | 75.4 |
|  |  | R | CCATCTGAAATTTTAATGATTCTA |  |  |
| chromosomal replication initiation protein | 469 | F | GCATGTTGTACTTTTGAC | 109 | 77.8 |
|  |  | R | CGTAGATTTAATGTTCCTATG |  |  |
| preprotein translocase subunit SecG | 476 | F | TGGCTGTTATCTGTTTAATTATTG | 114 | 77.6 |
|  |  | R | CCCTATCTTTGGTTTTACGAA |  |  |
| hypothetical protein MG_491 | 491 | F | ACCACTTAACTTTATTACCC | 84 | 76.3 |
|  |  | R | TGCGCATAATGAACTATG |  |  |
| formamidopyrimidine-DNA glycosylase | 498 | F | AGCAGCACAATTAAATAAAC | 172 | 77.0 |
|  |  | R | GCTTGCAAATAAGATTTCA |  |  |
|  | 527-v2 | F | ACTCCTTCTTATCAAGACA | 94 | 74.6 |
|  |  | R | CAGGTTGAAATTGTTGTTG |  |  |
| hypothetical protein MG_032 | 32 | F | TGCCTTGAGAGATGATAA | 170 | 78.6 |
|  |  | R | GTGGACTTAAAGTGAAACTA |  |  |
| putative nicotinate phosphoribosyltransferase | 37 | F | GTCCTTGATAACATTATTCCA | 141 | 76.6 |
|  |  | R | TGCTCATGTACTTTTATCTTG |  |  |
| spermidine/putrescine ABC transporter, permease protein, putative | 43 | F | GCGTTTGTTATTTCAATTG | 177 | 76.3 |
|  |  | R | AGGAGTAAACAGATAAATTG |  |  |
| spermidine/putrescine ABC transporter, permease protein, putative | 44 | F | ACCACTAATAAATTCAATCATTA | 163 | 77.3 |
|  |  | R | AGCAGTAATAATATCAGGAG |  |  |
| putative DNA-binding/iron metalloprotein/AP endonuclease | 46 | F | GCTACTATTATTGATCATTACATTG | 135 | 75.6 |
|  |  | R | GGGTAAATTTAATGTACTAATTCTG |  |  |
| signal recognition particle protein | 48 | F | GTCAGGAAATTATCAATGTTG | 115 | 77.5 |
|  |  | R | GAAGCTAATGAAAGTGCA |  |  |
| SsrA-binding protein | 59 | F | GACCTAAACTTAAAGCTTTA | 88 | 73.6 |
|  |  | R | AAGGCTAAATATGACTATCA |  |  |
| ABC transporter, ATP-binding protein | 65 | F | CCACATTACTAAACATTATTTCA | 98 | 74.2 |
|  |  | R | GAGCTTTCTATCATTTAAACAAA |  |  |
| putative lipoprotein | 68 | F | CCAGTTTAAAATTAACTCTATTC | 151 | 76.3 |
|  |  | R | CTCAGGGTTATTTTCATC |  |  |
| preprotein translocase subunit SecA | 72 | F | AGGAGTTTCTCAAGATTTATAAC | 176 | 79.1 |
|  |  | R | AGCAGTTCCAATCAGAATA |  |  |
| hypothetical protein MG_074 | 74 | F | CTTGCTTTTCTTAAGTGAAC | 95 | 73.9 |
|  |  | R | CAGTGTAGGAAAATACTGAA |  |  |
| oligopeptide ABC transporter, permease protein (OppB) | 77 | F | TGCCCAAAATATTGTGTTTA | 147 | 79.4 |
|  |  | R | CCAAGTAAGATAAAGTTAGCA |  |  |
| oligopeptide ABC transporter, ATP-binding protein | 80 | F | ACCACTTGTTAATGTTAAGG | 177 | 80.1 |
|  |  | R | CTAGGAATGTTTAATCTAATCAAA |  |  |
| hypothetical protein MG_096 | 96 | F | AGCCAATCCTTATAAATTCA | 146 | 79.4 |
|  |  | R | CGTTGACAACAAATGTTAA |  |  |
| hypothetical protein MG_103 | 103 | F | ACCAGTTAGTTGTTTATCTTAA | 198 | 76.8 |
|  |  | R | GGAGTTGATTAGTTTGATTATTAG |  |  |
| competence/damage inducible protein CinA | 115 | F | TGGCTTATTAGCTCATTG | 114 | 76.1 |
|  |  | R | GAGGATTGAACATTCAGTA |  |  |
| hypothetical protein MG_117 | 117-v2 | F | GGATGAATTTGATCAGATTAC | 84 | 75.2 |
|  |  | R | CTCCAATCATCAAAACTTTG |  |  |
| ABC transporter, permease protein | 120 | F | ACCCTAATACTGCTAGTTTA | 124 | 75.8 |
|  |  | R | GTGAGATTTATCTTTCCAATG |  |  |
| thioredoxin | 124 | F | TGTGGTCCTTGTAAACTA | 126 | 78.2 |
|  |  | R | AGAGGTAATGTTATAAGCTG |  |  |
| hypothetical protein MG_130 | 130 | F | AGTCCTATTAAAGCACAAA | 94 | 75.5 |
|  |  | R | GTTGGACATATGATTCAATC |  |  |
| hypothetical protein MG_134 | 134 | F | AGGAGATGATTATAGAAGCTA | 82 | 74.9 |
|  |  | R | GGCATAGTTTTCTCAACTAA |  |  |
| GTP-binding protein LepA | 138 | F | CCCATATTGATCATGGTAAA | 144 | 77.2 |
|  |  | R | CAGCATTTAATTTAATGGTAATAC |  |  |
| hypothetical protein MG_144 | 144 | F | ACCAGATGAAATAACAGAAA | 155 | 76.7 |
|  |  | R | ATGCCACAATAACTAAATTG |  |  |
| hypothetical protein MG_147 | 147 | F | GTGGGATTATTATCTACTTCTTTA | 100 | 76.7 |
|  |  | R | CCATGTAAAACAAACTACTAATG |  |  |
| cobalt transporter ATP-binding subunit | 179 | F | GCCATTAATTAGGGAACTATC | 117 | 76.6 |
|  |  | R | GGCTTTAATAACCCTGTTAA |  |  |
| ABC transporter, permease protein | 188 | F | ACGCAATAGTTTTCTCTATTC | 115 | 75.8 |
|  |  | R | CTGTCAAAACCCTCTTAACAA |  |  |
| co-chaperone GrpE | 201 | F | AACCCTTAAACATCATCG | 186 | 79.5 |
|  |  | R | CAGACATAATCTTTTCATCAAA |  |  |
| hypothetical protein MG_202 | 202 | F | ACCACTATGAAGAGTTACA | 109 | 77.1 |
|  |  | R | GCTACATAGTTAGATAAGATTAAC |  |  |
| hypothetical protein MG_233 | 233 | F | TCCCAAAACTTTCTAGTTG | 93 | 77.4 |
|  |  | R | CCCAAAGATAATTCCACTAA |  |  |
| hypothetical protein MG_236 | 236 | F | CCTTGATTTAGCCAATCA | 124 | 77.8 |
|  |  | R | CAGGCAATAGTAAGTTTCA |  |  |
| ATP-dependent protease La | 239 | F | CTCAGATGCAGATATTAATGA | 129 | 76.6 |
|  |  | R | CCTTCCATTAACTGTTCAA |  |  |
| hypothetical protein MG_241 | 241 | F | AAGCCTTTGATTATTCCAA | 84 | 75.8 |
|  |  | R | GGTTGATAACAGTTAAATTAGA |  |  |
| hypothetical protein MG_242 | 242-v2 | F | CTCAGAAGTTTCAGAAGTTTTC | 110 | 75.4 |
|  |  | R | CAAGCTCTTCTTCACTTAAAG |  |  |
| hypothetical protein MG_248 | 248-v2 | F | GCACAATTGCTAATTTAGTTC | 80 | 74.6 |
|  |  | R | GGATGTTAGATAGGAATGATC |  |  |
| RNA methyltransferase | 252 | F | GTTGCTTATTTGGTGTTAAAG | 179 | 76.2 |
|  |  | R | CTGGTGGTTAATATCTCTATAC |  |  |
| hypothetical protein MG_255 | 255 | F | TCTGGATTTAAGCAATGAA | 127 | 76.5 |
|  |  | R | TGCAGTAGTTAGATAATCAA |  |  |
| hypothetical protein MG_256 | 256 | F | GTAGCAATCTTTCTAATCTTTC | 131 | 76.0 |
|  |  | R | CTCACAATTAACCACAAGTTA |  |  |
| putative lipoprotein | 260-v2 | F | ATGGCTTGTAGTGTTGTA | 169 | 79.2 |
|  |  | R | CAGCAATTTGTACTATTTCAA |  |  |
| hypothetical protein MG_267 | 267 | F | ACCAGTCTTATTTAAAACAATG | 102 | 76.1 |
|  |  | R | AAGCGATAGAGTATGTCAA |  |  |
| hypothetical protein MG_268 | 268 | F | GATCGTTCTATCTTTGAAGA | 83 | 76.9 |
|  |  | R | CACAGTTGGTTATAGTATGAA |  |  |
| hypothetical protein MG_269 | 269 | F | AACGCCAACAAATTATCA | 148 | 79.0 |
|  |  | R | AACCCTGTTAATAGAATTGG |  |  |
| hypothetical protein MG_277 | 277-v2 | F | CTCTCACCTTTGAACAAA | 111 | 79.4 |
|  |  | R | GTTGGTAGTTGTGTTCAA |  |  |
| hypothetical protein MG_279 | 279 | F | CCCAAATCCAAACCAAAA | 143 | 76.0 |
|  |  | R | CTCCATAAACTTTCTTTGTAAA |  |  |
| hypothetical protein MG_280 | 280 | F | GGTGTAATTAGTGCAGTTA | 174 | 77.8 |
|  |  | R | GGTTGTAATCAAAATTGCTAA |  |  |
| hypothetical protein MG_281 | 281 | F | GCAGAAGTAAAGTTGAAATC | 125 | 77.4 |
|  |  | R | ATCGCTTCTGATAACTCA |  |  |
| transcription elongation factor GreA | 282 | F | CAGGGTGATTTAAGTGAAA | 100 | 77.3 |
|  |  | R | CGTTGGCTAATATATCTTGA |  |  |
| hypothetical protein MG_285 | 285 | F | GGAAGTTATGAAATAATGATTGG | 171 | 76.8 |
|  |  | R | CTGAGTTAAATCACTAAAGTTG |  |  |
| hypothetical protein MG_286 | 286 | F | GCAACATAACTATTTCCAAC | 164 | 76.6 |
|  |  | R | GCTCAAATTGATCTCAATATC |  |  |
| phosphonate ABC transporter, substrate binding protein (P37), putative | 289 | F | GCTTCAAATAATTGGTTTGG | 92 | 75.2 |
|  |  | R | CCATCTTCATAATAATCAAGC |  |  |
| phosphonate ABC transporter, permease protein (P69), putative | 291-v2 | F | GATGCTTATGATAGTGTAATTAG | 116 | 74.8 |
|  |  | R | GAACCTAGATTATCAAATTGAG |  |  |
| major facilitator superfamily protein, putative | 294 | F | GCTACTGTTACTTCTATCATTG | 144 | 77.6 |
|  |  | R | GTTGGCTGCTAATCAAAA |  |  |
| hypothetical protein MG_296 | 296 | F | CGAGAACAAGACAATATTG | 118 | 74.8 |
|  |  | R | CTAGCTGTTTAATTACTTCAG |  |  |
| signal recognition particle-docking protein FtsY | 297 | F | CTGTCAAAACGATATGTTAA | 127 | 77.0 |
|  |  | R | GCTTGATCTGTTCAATAATAG |  |  |
| metal ion ABC transporter, permease protein, putative | 302 | F | CAGGACAGTTGTTTACAA | 143 | 76.2 |
|  |  | R | TGCAGTATATGCATGAAC |  |  |
| molecular chaperone DnaK | 305 | F | CGATCCTTTGTAATTGCATTA | 73 | 75.8 |
|  |  | R | GGTTGAACCTTAATATGAAATAAC |  |  |
| putative lipoprotein | 307-v2 | F | GATGCTTTATCAATCCCATA | 187 | 77.6 |
|  |  | R | CTGGTTTAAATGTTGAAAGG |  |  |
| alpha/beta fold family hydrolase | 310-v2 | F | GTTCCATGTTAGAAAACAATAC | 120 | 77.4 |
|  |  | R | TTGCCATTACTACTACTACTA |  |  |
| hypothetical protein MG_313 | 313-v2 | F | GCAGGATGGAATTATTAAGTA | 105 | 77.3 |
|  |  | R | TGTGGTTGTTCAAAGTAAG |  |  |
| hypothetical protein MG_314 | 314-v2 | F | TGTGGTTTTGGATGTTTG | 153 | 77.6 |
|  |  | R | CAACCTTGTAATTACTGGC |  |  |
| HMW3 cytadherence accessory protein | 317 | F | CCTGGTAATATAGTAAGAAGTG | 99 | 74.8 |
|  |  | R | GATGCTAAAAGGAACAATG |  |  |
| hypothetical protein MG_319 | 319-v2 | F | GCTAGAACTAAATGGTAAACTA | 108 | 76.0 |
|  |  | R | CCATGAAGATTAACTAATATCAG |  |  |
| hypothetical protein MG_320 | 320 | F | AGTGCATTTTATGCTATCC | 112 | 76.7 |
|  |  | R | TAGGGGAATTACATACTTAGTTA |  |  |
| putative lipoprotein | 321 | F | CCGTAATGAACAAGATTTG | 178 | 79.2 |
|  |  | R | GAAGCTGCTAATTAATCAAG |  |  |
| TrkH family potassium uptake protein , putative | 322 | F | CCACATTGGTTATGAATTG | 135 | 77.0 |
|  |  | R | AAGCGAAGGAATAGTTTC |  |  |
| TrkA family potassium uptake protein | 323 | F | CTCACTAGTATTACCTAGCTTA | 97 | 77.0 |
|  |  | R | GGGATTGGATTTGTTGTTA |  |  |
| hydrolase, alpha/beta fold family | 327 | F | CTGCTTTAGAAGAGTTACAA | 109 | 75.8 |
|  |  | R | TCCCTTAAATTAGCACATATC |  |  |
| hypothetical protein MG_328 | 328-v2 | F | CTACCTTAGTTATTTTAGGAG | 70 | 74.9 |
|  |  | R | ACACCATCAATAACCTTAA |  |  |
| GTP-binding protein EngA | 329 | F | CTGGTTTTCAGGAACTAAA | 165 | 76.6 |
|  |  | R | GCTTCTGGTTTTATCTCA |  |  |
| cytidylate kinase | 330 | F | GCAACAACTAATTGCATTAC | 119 | 73.9 |
|  |  | R | TCAGCTTTATTTACCACTAG |  |  |
| putative lipoprotein | 338 | F | GCAAGTGAATAGATTAAATATTGA | 153 | 77.0 |
|  |  | R | GGGTTGTAATAACTGTTGTAA |  |  |
| RNA methyltransferase | 346-v2 | F | GCAGTTAGAATGGAATCAA | 188 | 77.2 |
|  |  | R | ACCTCCATCAATATGAAATG |  |  |
| putative lipoprotein | 348 | F | TGTCCAGAAATTCCTAATAAC | 86 | 75.8 |
|  |  | R | GTTGCTTGTAAGAAATGTTC |  |  |
| ImpB/MucB/SamB family protein | 360 | F | TGGACAAAATACTACTCCTA | 152 | 76.0 |
|  |  | R | CGTGTTGTTTAATTCCATAAG |  |  |
| hypothetical protein MG_364 | 364 | F | GAGAGTTGTAATCTTATAAAGAG | 107 | 76.4 |
|  |  | R | CTAGGCAAATGATATACAGATA |  |  |
| hypothetical protein MG_366 | 366-v2 | F | GAAGCTGAGGTTTATCAAA | 113 | 73.8 |
|  |  | R | GGTTGAATTCATTGCATTTC |  |  |
| uridine kinase | 382 | F | GCGTTTTCTAAATTGCTTAA | 130 | 74.2 |
|  |  | R | CTTGGATAAATAAAACAATTGAAA |  |  |
| GTPase ObgE | 384 | F | GTTGCTGAAATGATTTATCAA | 110 | 75.2 |
|  |  | R | CAGGATGATCAAAGTTTATTG |  |  |
| P200 protein | 386-v3 | F | CGTCTAAGATATTGATTTGATAA | 169 | 75.9 |
|  |  | R | CCAACGGATTTTAATTGATC |  |  |
| putative lipoprotein | 395-v2 | F | AGCAGTTATCCAACCATTA | 171 | 77.0 |
|  |  | R | CACCAACAAAGATATATCAGAA |  |  |
| hypothetical protein MG_406 | 406 | F | ACACCAAAATAAATACTGTTAA | 137 | 75.6 |
|  |  | R | AGGGATTGTTACATTTCAAA |  |  |
| OsmC-like protein | 427 | F | GTAGCTTGGTTATTTGTACTA | 110 | 77.2 |
|  |  | R | GATGCTAATCTTAAAACTGATAG |  |  |
| putative lipoprotein | 439 | F | TGGTGATTCAAGATATGGA | 81 | 73.9 |
|  |  | R | TCTGGATTAAATTTTATTAAAGCA |  |  |
| putative lipoprotein | 440-v2 | F | GATGCTTACAAAGGAAGAA | 146 | 74.0 |
|  |  | R | ACTGCTAATTTGCCTAAA |  |  |
| degV family protein | 450-v2 | F | AGCTGCTAAGATGATAAC | 120 | 75.5 |
|  |  | R | CTCAGTGAATGTTAAGTTC |  |  |
| hypothetical protein MG_459 | 459 | F | TGGTGATGTTATCTTTACTTA | 149 | 76.4 |
|  |  | R | GAAGCAGGTATACTACTAAA |  |  |
| L-lactate dehydrogenase/malate dehydrogenase | 460 | F | TCAGCTAATTGACCTACA | 157 | 76.8 |
|  |  | R | CCTTCCAGTAATTTATTTTCC |  |  |
| HD domain-containing protein | 461 | F | AGCAGAATAGTTGAAGTTTTAATAG | 174 | 77.2 |
|  |  | R | GCACTTATTACGGAAGTTATAAAC |  |  |
| putative inner membrane protein translocase component YidC | 464 | F | AGTCCAATTTAAAGGTACTG | 196 | 78.0 |
|  |  | R | TAGCGTGTTTATAATCATCAA |  |  |
| ABC transporter, permease protein | 468 | F | GGTAGTGAATACATGATTATAGA | 172 | 77.4 |
|  |  | R | CTGCACTAATGAAAATAAAATC |  |  |
| hypothetical protein MG_477 | 477 | F | GAACCTATTGTCTCTATCTTAA | 177 | 78.4 |
|  |  | R | ACCTGTAAAATAAGCATCA |  |  |
| hypothetical protein MG_515 | 515 | F | CGCAAATAGTTTTACCAATTATG | 97 | 75.4 |
|  |  | R | GCGTTAATTTCTGCTAATTTAC |  |  |
| ABC transporter, ATP-binding protein | 526-v2 | F | GAACCAATTACAAAGATAGTAAC | 96 | 74.2 |
|  |  | R | CTGCATTTGTTTTAGCATTA |  |  |
| 30S ribosomal protein S9 | 417 | F | CAACCAACTTGAAATTGATC | 151 | 78.0 |
|  |  | R | TGTGCTAATTGTTGTAAGG |  |  |
| 50S ribosomal protein L13 | 418 | F | TGGTGGTTGGATTAATAAGA | 84 | 76.8 |
|  |  | R | GCTGTAAAACAGATAGTTACTG |  |  |
| glyceraldehyde-3-phosphate dehydrogenase - MG GAPDH | 301 | F | GGTTGTTAATGCCATTTG | 166 | 78.2 |
|  |  | R | ACCCCAAAAGAAAATCAC |  |  |
|  | MG 16s | F | AACCTTACCTAGACTTGACATCCT |  | 81.5 |
|  |  | R | TTGCGGGACTTAACCCAACA |  |  |

Shaded boxes indicate primers that may form primer-dimers in reactions with little to no template.
