# Supplementary material for: Development and Utilization of a Custom PCR Array Workflow: Analysis of Gene Expression in Mycoplasma genitalium and Guinea Pig (Cavia porcellus)
Source: Mol Biotechnol. 2014 Oct 31;57(2):172–83. doi: 10.1007/s12033-014-9813-6 (PMC4298676; doi:10.1007/s12033-014-9813-6)
Supplement: Supplementary file 2 — Supplementary material 2 (DOC 280 kb) [file 12033_2014_9813_MOESM2_ESM.doc]

**Supplementary Table S2** List of primers included on the gpArray

| **Gene** |  | **Sequence (5’ – 3’)** | **Length (bp)** | **Amplimer T_m_ (°C)** |
| --- | --- | --- | --- | --- |
| TLR-2 | F | GTGCATATTCCAAACTTCTA | 83 | 75.6 |
|  | R | TCCAGTGTGATTCGTTTA |  |  |
| CD4 | F | AGACCTTGAACCTGATTG | 174 | 84.8 |
|  | R | ACTGGAGATACTTCTTGTC |  |  |
| CD126 | F | CCAACATCATTGTCACTG | 92 | 81.8 |
|  | R | CCTGTAGAAGTCTGAGTTC |  |  |
| CD94 | F | TGGAGGATAATTTCTGGAA | 97 | 75.8 |
|  | R | GACTCATCAAGAGACACTTA |  |  |
| CD115 | F | GGAAGATCATTGAGAGCTA | 80 | 80.6 |
|  | R | CTCCCACTTCTCATTGTA |  |  |
| KLRG1 | F | GTGCCAGTGACATTTATTTC | 73 | 78.2 |
|  | R | TGCTGTGGTCTGTAGTTA |  |  |
| NFκB1 | F | CTCCGAAGTATAAAGACATCA | 82 | 79.8 |
|  | R | CTCGTTTCCAAGTCTGAC |  |  |
| TNFSF4 | F | TTGGCTTACAAAGACAAA | 96 | 79.6 |
|  | R | CTGGATGAGATACAGTTC |  |  |
| SOD1 | F | GAGCAAATTCCATCATTG | 89 | 80.5 |
|  | R | CGTCTTTGTACTTTCTTCA |  |  |
| IL-23 receptor | F | GACACCAACTTCACATAC | 74 | 79.4 |
|  | R | CGCACTTGAAATACGTAA |  |  |
| CCR3 | F | CCAACATTTACCTGCTCAA | 82 | 79.7 |
|  | R | CAGTGAACATAGTAAGTCCA |  |  |
| CXCR1 | F | TGGTCAAGTTCATCTGTA | 115 | 81.4 |
|  | R | GACTCATAGCAGACAAAG |  |  |
| TLR-3 | F | GGTCCTGTTCATTTTCTAA | 126 | 78.6 |
|  | R | CCCTAAATTGATGCTCTTAA |  |  |
| CD8α | F | GGAACAGAAGCGATTCAG | 100 | 82.6 |
|  | R | GACCGAGCAGAAGTAGTA |  |  |
| CD130 | F | CCACCTCGTAATTTATCAG | 96 | 75.8 |
|  | R | CAGTCTCATAACATTCATAATAC |  |  |
| CD23 | F | CCCTGAAGAACGTTAAAC | 90 | 81.0 |
|  | R | TCTGGTTACTGTGATGTC |  |  |
| CD2 | F | AGACCTGAAATTGTACTTCA | 83 | 78.6 |
|  | R | TGCAGATGTAGCAAATGA |  |  |
| β_2_ microglobulin | F | AGTCGAATTGCTGAAGAA | 71 | 77.0 |
|  | R | AGTCCTTGCTGAAAGAAA |  |  |
| CCL20 | F | GACTGTTGTCTCAGATACA | 129 | 81.8 |
|  | R | CACAGATAGTCTCTTCTTTG |  |  |
| CCR6 | F | CTTGCCAGATACCTCATAA | 176 | 84.6 |
|  | R | GAACCTTTGACCGATAAAC |  |  |
| IFNAR2 | F | GAAGGTATGAAGATTGTGAAG | 72 | 76.8 |
|  | R | TTGCCACACATCTGTTAG |  |  |
| IL-27 | F | CTGCTCTACACCTATCAG | 81 | 82.3 |
|  | R | CTTGGACAGCAGTAGTAA |  |  |
| IL-8 | F | CCCAAATTTATCAAAGAACTG | 77 | 76.9 |
|  | R | CTGAGTTTCACAATGATTTC |  |  |
| CXCR3 | F | TCTCCTTACGACTATGGA | 84 | 81.8 |
|  | R | GTCGAAGTTTAGGCTGAA |  |  |
| TLR-4 | F | CCTTCACTACAGAGACTTTA | 146 | 82.3 |
|  | R | GAGCAATCTCATATTCAAAGA |  |  |
| CD62 ligand | F | GTACCAAAACCACAACAA | 121 | 86.4 |
|  | R | CACAGTGACGTAGTAAAC |  |  |
| CD19 | F | CCACTGAGATACACATGA | 110 | 83.8 |
|  | R | GCAGAAGATCAGATAAACC |  |  |
| CD25 | F | CCCATAATGCAAGAGAAC | 87 | 77.9 |
|  | R | TAGGCTGTATCTGACTTTG |  |  |
| IFNγ | F | CCATCAAGGAACAAATTATTAC | 90 | 77.7 |
|  | R | TGACCGAAATTTGAATCAG |  |  |
| MHC-II | F | GGATCATGTGTCAACATTTG | 77 | 78.7 |
|  | R | CCTCATCAAGCTCAAACATA |  |  |
| IL-15 | F | GGCAAATAGCAGTTTAAATTC | 70 | 74.9 |
|  | R | CTCCAGTTCTTCACATTC |  |  |
| CD36 | F | GGATGTTTACAGACAGTTC | 89 | 79.2 |
|  | R | CTCTCTGCTTAACCTTTATG |  |  |
| CD96 | F | GCACTCAGGAAACTATTATC | 91 | 78.4 |
|  | R | AGTCGATAGCTTGTATCTAG |  |  |
| IL-27 receptor α | F | GTGGACTTCTCAGAGGAC | 100 | 85.6 |
|  | R | TCTGGTAGTAGAACTGACAG |  |  |
| SOD2 | F | GGAACAACAGGTCTTATTC | 98 | 77.7 |
|  | R | ATGGCTTTTAGATAATCAGG |  |  |
| CXCR2 | F | CTGAGCCAAATTTCATCA | 70 | 77.1 |
|  | R | CTACGACATAAGTATTGATTTC |  |  |
| TLR-6 | F | CTCACTTGAACCTAAATTAC | 85 | 75.0 |
|  | R | GTCAGAATTTGAAGATTCTC |  |  |
| CD107a | F | CAGAGTGGTCAACATCAA | 91 | 83.4 |
|  | R | GCTCTCTTCACTCTTCAG |  |  |
| CD22 | F | CTCCTACCTTCAGAAATAAAG | 82 | 78.4 |
|  | R | GGATGTCATACTCAAAACA |  |  |
| CD39 | F | TGCTCTCAAAATATCCTG | 98 | 80.2 |
|  | R | TCAGGTAATGGTTTGTTC |  |  |
| IFNAR1 | F | CTGAGATGGATAATTGGATAA | 150 | 77.4 |
|  | R | CAACGTAATACCATGAAGA |  |  |
| CIITA | F | AGCGAAATCAAGGACAAG | 82 | 75.8 |
|  | R | CTTCCATCCAGTTGTCATA |  |  |
| CXCL10 | F | GCCACAATGAAAATGAATG | 76 | 70.6 |
|  | R | CTGCTTTCAGTAAATTCTTAATG |  |  |
| CD180 | F | CTACCAAACACAACAGAA | 72 | 73.6 |
|  | R | GCTGAAGGTTATATCTTGAA |  |  |
| IL-12p40 | F | TCCAGGTCAAAGAGTTTG | 114 | 81.2 |
|  | R | CAGTGGACCAAATTTCATC |  |  |
| CTSG | F | CCTCTGGTATGTAACAATG | 82 | 78.7 |
|  | R | TCCTGGTAAAGACTTCTG |  |  |
| TGFβ | F | CACAGTATATATATGTTCTTCAAC | 110 | 83.9 |
|  | R | TCCACATTTAACTTGAGTC |  |  |
| IL-5 receptor β-chain | F | ATGGGGAGAAATTCATAAG | 128 | 83.3 |
|  | R | CTCGATGTGGGAATAGTA |  |  |
| TLR-7 | F | GCTGAAATACTTAGACTACTC | 123 | 79.0 |
|  | R | CCTTCTGATTGAAAATAATGG |  |  |
| CD107b | F | GCTTCAGTTATTAACATCAAC | 90 | 79.0 |
|  | R | GTCACTGTTATTCAGCTTAA |  |  |
| CD79a | F | ATGGCAACAACTCCAAAG | 126 | 83.8 |
|  | R | GACTCTTGTTCACATTGGA |  |  |
| CD69 | F | CTAGCTTCCGTTTTGAAA | 106 | 79.2 |
|  | R | GCGATGACAGTAATGAATA |  |  |
| IFNGR1 | F | CCGAAATGGTTCCGATAG | 101 | 80.2 |
|  | R | CCTGTCCTTCTGTCTTTA |  |  |
| RANTES | F | CAAGGAATATTTCTACACCA | 128 | 81.6 |
|  | R | TCTCCAAAGAGTTGATGTA |  |  |
| CXCL11 | F | GCTTCCCTATGTTCAAAA | 100 | 79.1 |
|  | R | CTTGGGTAAATTATAGAGGC |  |  |
| CD28 | F | ATGGGAATTTGGACAATG | 88 | 79.2 |
|  | R | ACCTCAATTTTGCAGAAG |  |  |
| IL-4 receptor | F | CAACCTGACCTACAAGGA | 123 | 85.6 |
|  | R | CTCCACTCACTCCAGATG |  |  |
| CD14 | F | CCTGTCCTTGAAAGGAAA | 82 | 81.0 |
|  | R | CACGTTAGACTCAGAGTTC |  |  |
| TNFα | F | GGAAGAGCAGTTCTCCAG | 89 | 83.3 |
|  | R | GCTTGTCATTATCGTTTTGAG |  |  |
| GAPDH | F | CTCGTCATCAATGGAAAG | 98 | 83.6 |
|  | R | GTGGATTCCACTACATAC |  |  |
| TLR-8 | F | GTCTGGGATTTCTTGAAA | 87 | 75.4 |
|  | R | GCTCATTTTCCTCTGTTA |  |  |
| CD134 | F | GCTGGTTTCAGAGAGAAG | 100 | 83.4 |
|  | R | AGGAGAAAGAAGGTCACA |  |  |
| CD79b | F | GCGGAATACACTGAAAGA | 72 | 77.6 |
|  | R | ATGGGCACAATGATGAAA |  |  |
| CD72 | F | ACGCTGCTTTTACTTTTC | 75 | 76.2 |
|  | R | GTGGATGACAGAGATGTA |  |  |
| GM-CSF | F | ATGCCACCATCAATGAAG | 90 | 81.5 |
|  | R | GGTCATAGACAACTTCTACTG |  |  |
| MCP-1 | F | AGGGTTATGAAAGAATCAC | 78 | 79.2 |
|  | R | AGACCTCCTTGTTCTTTA |  |  |
| IL-12p35 | F | CACTGGAATTAGTCAAGAA | 132 | 81.8 |
|  | R | AGTCCTCATAGATACTGTTA |  |  |
| CD40 | F | TACGGCTACTTCTGATAC | 84 | 79.5 |
|  | R | GGGTGACACTTTTCAAAA |  |  |
| IL-7 | F | GATCCTTGTTCTGTTACC | 91 | 75.2 |
|  | R | GATGCTGACCAGTATAAC |  |  |
| IL-7 receptor | F | GACGCAATGTATGAGATTA | 102 | 79.7 |
|  | R | CTCTGGAGTTTTGAAGTG |  |  |
| BPI | F | TCCTGAGTTTCCATCTTC | 95 | 82.0 |
|  | R | CCCATTCATAACGACATTG |  |  |
| HPRT1 | F | GACCTAGATTTATTTTGTATTCC | 81 | 74.2 |
|  | R | GTCCATAATTAGTCCATGAG |  |  |
| TLR-9 | F | CACTCAAGTATAACAATCTTAC | 99 | 82.4 |
|  | R | CCGATTCCAGTTTAATAATG |  |  |
| CD152 | F | CCAGATTCTGACTTCCTC | 76 | 79.2 |
|  | R | CTGTGATGAGGAAACTGTA |  |  |
| CD20 | F | TCCCGTGACATATATTAACA | 94 | 77.8 |
|  | R | GGACTGTATGGTGTAACA |  |  |
| CD92 | F | TCCTGATAATGCTGGTTA | 102 | 81.5 |
|  | R | GTTGGAAGACTAACAGTG |  |  |
| LTA | F | CCTCAATGGCTTCTCTTTG | 71 | 80.6 |
|  | R | GCGAGTAGACAAAGTACAG |  |  |
| MCP-3 | F | AGACCAAATTTAACAGAGAAA | 91 | 77.9 |
|  | R | GGAGTTTTGGATTTCTTATCTA |  |  |
| GNCP-1b | F | GCTGTATTTGCACAACAA | 73 | 79.6 |
|  | R | ACTCGATTCTGGAAGATG |  |  |
| CD44 | F | AGGAGAATACAGAACACA | 115 | 81.4 |
|  | R | GCCATAAGTGCTTCTTTC |  |  |
| IL-17α | F | AAGGCAGGAATACCAATC | 111 | 80.6 |
|  | R | TTGGGTAAGAGGATTGAAG |  |  |
| IL-18 | F | CCTCCTGATAATATCAATGAC | 107 | 76.2 |
|  | R | TAGCCTTTATACAATGAAGAC |  |  |
| CXCL12 | F | CAGCCTGAGTTACAGATG | 86 | 82.0 |
|  | R | GAGTGTTGAGGATTTTGAG |  |  |
| β-actin | F | CTACCTTCAACTCCATCA | 166 | 84.6 |
|  | R | GGAGCAATGATCTTGATC |  |  |
| TLR-10 | F | TGGGTAAAGAATGAATTGG | 117 | 78.8 |
|  | R | GCAGTTAATGATGTTTTCAG |  |  |
| CD223 | F | CAGGAGTCCTCACATCAC | 104 | 82.2 |
|  | R | CCATCTCTGTAAGTAAGAATGC |  |  |
| CD30 | F | CACAGTCCTGTCCTCAG | 79 | 82.2 |
|  | R | TAGCCGCTCTCATCTAC |  |  |
| CD93 | F | TGCTGCTGTTCTACATC | 120 | 86.6 |
|  | R | GTGGCTTCTTCTCCTTTA |  |  |
| Lysozyme | F | TGGGAGAGTGATTATAATACA | 86 | 76.8 |
|  | R | CGACTATTGATCTGGAATATC |  |  |
| F_c_ γ1/γ2 receptor | F | CGCTAAATCTGAGGTTGA | 95 | 79.4 |
|  | R | TGTGGTTCTGATAGTCATG |  |  |
| IL-1β | F | CACAGTGGAATTTGAATCC | 129 | 80.8 |
|  | R | GACACTAGTTCTAACTTGAAG |  |  |
| CD81 | F | GCTCCAACACATTGATTG | 89 | 81.8 |
|  | R | CTTCCATAAGGTATTTATGAAGG |  |  |
| IL-16 | F | GGCTGAAGAAGTCTTTTG | 86 | 80.4 |
|  | R | GTCGCTTTTGAAGTATTTTC |  |  |
| IL-21 | F | AAGGCTCAACTCAAGTTA | 76 | 76.2 |
|  | R | TCCTCTTCAACTGCTTAA |  |  |
| CCR4 | F | TGAGGACCTTTACGTATG | 187 | 84.8 |
|  | R | CCTAGAATGTTGATCTCCA |  |  |
| eEF1a1 | F | TGGGTGTGAAACAACTAA | 99 | 79.4 |
|  | R | TGCTGACTTCCTTAACAA |  |  |

Shaded boxes indicate primers that may form primer-dimers in reactions with little to no template.
